# Variability in Characterizing *Escherichia coli* from Cattle Feces: A Cautionary Tale

**DOI:** 10.3390/microorganisms6030074

**Published:** 2018-07-21

**Authors:** Kim Stanford, Tim Reuter, Jennyka Hallewell, Renata Tostes, Trevor W. Alexander, Tim A. McAllister

**Affiliations:** 1 Alberta Agriculture and Forestry, Lethbridge, AB T1J 4V6, Canada; tim.reuter@gov.ab.ca; 2 Agriculture and Agri-Food Canada, Lethbridge, AB T1J 4B1, Canada; jennyka.hallewell@agr.gc.ca (J.H.); trevor.alexander@agr.gc.ca (T.W.A.); tim.mcallister@agr.gc.ca (T.A.M.); 3 JBS Food Canada Inc., Calgary, AB T2H 1M7, Canada; renata.tostes@gmail.com

**Keywords:** Shiga toxin-producing *Escherichia coli*, *stx* detection, serotype, sub-cultures

## Abstract

Shiga toxin-producing *Escherichia coli* (STEC) are diverse bacteria, with seven serogroups (O26, O45, O103, O111, O121, O145, O157; “Top 7”) of interest due to their predominance in human disease. Confirmation of STEC relies on a combination of culturing, immunological and molecular assays, but no single gold standard for identification exists. In this study, we compared analysis of STEC between three independent laboratories (LAB) using different methodologies. In LAB A, colonies of Top 7 were picked after serogroup-specific immunomagnetic separation of feces from western-Canadian slaughter cattle. A fraction of each colony was tested by PCR (*stx1*, *stx2*, *eae*, O group), and Top 7 isolates were saved as glycerol stocks (*n* = 689). In LAB B, a subsample of isolates (*n* = 171) were evaluated for *stx1* and *stx2* using different primer sets. For this, approximately half of the PCR were performed using original DNA template provided by LAB A and half using DNA extracted from sub-cultured isolates. All Top 7 isolates were sub-cultured by LAB A and shipped to LAB C for traditional serotyping (TS) to determine O and H groups, with PCR-confirmation of virulence genes using a third set of primers. By TS, 76% of O groups (525/689) matched PCR-determined O groups. Lowest proportions (*p* < 0.05) of O group matches between PCR and TS (62.6% and 69.8%) occurred for O26 and O45 serogroups, respectively. PCR-detection of *stx* differed most between LAB A and LAB C. Excluding isolates where O groups by PCR and TS did not match, detection of *stx1* was most consistent (*p* < 0.01) for O111 and O157:H7/NM. In contrast, for O45 and O103, *stx1* was detected in >65% of isolates by LAB A and <5% by LAB C. *Stx2* was only detected by LAB C in isolates of serogroups O121, O145, and O157:H7/NM. LAB B also detected *stx2* in O26 and O157:H12/H29, while LAB A detected *stx2* in all serogroups. Excluding O111 and O157:H7/NM, marked changes in *stx* detection were observed between initial isolation and sub-cultures of the same isolate. While multiple explanations exist for discordant O-typing between PCR and TS and for differences in *stx* detection across labs, these data suggest that assays for STEC classification may require re-evaluation and/or standardization.

## 1. Introduction

Among zoonotic pathogens, Shiga toxin-producing *Escherichia coli* (STEC) are a diverse group of bacteria, with seven serogroups (O26, O45, O103, O111, O121, O145, O157) of particular interest due to their predominance in human disease [1]. Of these “Top 7”, serotype O157:H7/NM is the most characterized and can be detected in concentrations as low as 25 CFU/g by direct plating on selective media after immunomagnetic separation (IMS) [2]. In contrast to O157, detection and enumeration of the other members of the Top 7 is less efficient, requiring molecular methods, as selective media for these other serogroups has yet to be developed [3,4]. Without the benefit of differential media, cross-reactivity in serogroup-specific IMS kits also increases the difficulty of isolating non-O157 STEC [5].

Numerous PCR-based assays have been used to confirm serogroup and virulence genes carried by STEC [6,7,8,9,10,11], although results can differ depending on assays used [12]. For decades, traditional serotyping (TS) based on O- and H-antigens and agglutination of antisera has been invaluable for distinguishing strains during outbreaks [13]. However, this process necessitates the generation of specific sera, is labor-intensive and is only performed by a few specialized laboratories. Even with the required resources, some strains of STEC are untypeable by this procedure [14]. Molecular serotyping of *E. coli* has shown promise [15], although isolates with up to 99.9% similarity of O antigen gene clusters may not type together by TS due to post-translational modification of proteins [16]. Diversity in *wzx* (O-antigen flippase) and *wzy* (O-antigen polymerase) gene clusters is the foundation for PCR-based identification of *E. coli* O-groups [9,10]. However, Shridhar et al. [17] demonstrated that a PCR assay targeting O104 *wzx* also detected serogroups O8 and O9, necessitating an additional assay to verify detection of O104. Classification of *E. coli* is continuously being refined, as illustrated by DeBroy et al. [16] advocating merging a number of *E. coli* O groups due to TS cross reactivity and homology between gene sequences, while others have proposed that there are likely undiscovered O groups [18].

Genetic plasticity of *E. coli* is another concern, influencing detection of STEC and complicating separation of STEC from non-pathogenic *E. coli.* Using PCR analyses, Joris et al. [19] reported a lack of detection of both *stx1* and *stx2* in STEC after sub-culturing, leading to possible underestimation of STEC-positive samples. Recently, Loftsdottir et al. [20] described a mobile insertion sequence element in *stx2*, which if present would interfere with PCR-based detection. In addition, the presence of *stx*-carrying bacteriophages that are not integrated into the bacterial genome may also interfere with PCR detection of Shiga toxins [21]. To determine the collective impact of factors that may influence characterization of STEC by O-group and *stx* gene detection, Top 7 isolates were sub-cultured and subjected to TS, pulsed-field gel electrophoresis and different methodologies of PCR in three laboratories.

## 2. Materials and Methods

### 2.1. PCR Confirmation of Serogroup and stx at Initial Isolation by Laboratory A (LAB A)

Isolates of Top 7 were collected from feces of western-Canadian cattle just prior to slaughter over a two-year period [22]. To collect these isolates, serogroup-specific IMS was performed using RapidChek Confirm STEC Kits (Romer Labs Technology Inc., Union, MO, USA) and incubating cultures on MacConkey agar at 37 °C for 18–24 h. Approximately half of a colony was then suspended in 40 µL 1 × TE buffer (10 mM Tris, 1 mM EDTA, pH 8.0). The suspension was heated to 95 °C for 5 min and used as DNA template for serogroup confirmation by PCR [10]. PCR conditions included 50 nM O121; 40 nM O103, O111, O145, and O157; 25 nM O26 and O45 primers; 1× QuantiFast Master Mix (Qiagen, Toronto, ON, Canada), 2 µL DNA template and nuclease-free water, all in a final volume of 25 µL. Thermocycling conditions included an activation step of 95 °C for 5 min followed by 45 cycles of 95 °C for 45 s and 66 °C for 60 s. The remainder of a colony confirmed positive by PCR was removed from the plate and sub-cultured at 37 °C overnight in tryptic soy broth (TSB) before storage in glycerol at −80 °C.

A loop of inoculum from glycerol stocks was grown overnight in 10 mL TSB at 37 °C before extraction of DNA using the NucleoSpin Tissue Kit (Machery-Nagel, Islington, ON, Canada). Shiga toxin (*stx1, stx2*) and intimin (*eae*) genes were detected using multiplex PCR, with the plasmid copy number regulating gene (*repA*) used as an internal control [10]. Reaction mixtures contained 20 nM of each primer, 1× QuantiFast Master Mix, nuclease-free water and 2 µL DNA template in a 25 µL total reaction volume that was subjected to the previously-described thermocycling conditions. The same methodology was also used to reconfirm Shiga toxin profiles for a subsample of isolates after two or more years of storage in glycerol stocks at −80 °C.

### 2.2. PCR Methodology Used for Detection of stx Genes by Laboratory B (LAB B)

Only isolates of O26, O103, O111, and O157 serogroups (*n* = 171) were evaluated by LAB B due to time and labor constraints. The original DNA template extracted using NucleoSpin Tissue kits by LAB A was used for approximately 50% of isolates from each serogroup, while for the remaining half, glycerol stocks were re-grown a second time and DNA was extracted using NucleoSpin kits. Both sources of DNA were used as insufficient original extracted DNA was available to complete all LAB B studies. Briefly, 1 µL of DNA template was added to 24 µL PCR mix containing 2× PyroMark Master Mix (Qiagen) and primers as described by Goji et al. [11]. PCR amplification was performed using a peqSTAR 96 Universal Gradient thermocycler (PeqLab, Erlangen, Germany), with the hotstart program starting at 95 °C for 15 min, 45 cycles at 94, 56 and 72 °C for 30 s each and final extension at 72 °C for 5 min.

### 2.3. Serotyping of Isolates and Detection of stx Genes by Laboratory C (LAB C)

Cells from glycerol stocks were grown a third time overnight in 10 mL TSB at 37 °C at LAB A before preparation of slants and shipment of isolates to LAB C, a national reference laboratory for STEC (Laboratory for Foodborne Zoonoses, Public Health Agency of Canada, Guelph, ON, Canada). Somatic (O) and flagellar (H) antigens were identified by accredited methods using reference antisera (SSI Diagnostica, Copenhagen, Denmark).

For detection of *stx* genes by PCR, bacteria were grown overnight at 37 °C in 1 mL Brain Heart Infusion Broth (BHI). A 6.25 µL sample of the overnight BHI culture was added to 50 μL of lysis buffer and incubated at 60 °C for one hour and then 97 °C for 15 min. From this DNA template, primer sets and conditions described by Lin et al. [7] were used to detect undifferentiated *stx* (*stx1* and *stx2*) simultaneously in a singleplex PCR. Additionally, the DNA templates were used for the multiplex PCR assay of Paton and Paton [8] which also included an additional primer for detection of *stx2*f [12]. In all three laboratories, highly-experienced staff performed the PCR.

### 2.4. Pulsed-Field Gel Electrophoresis (PFGE)

A subsample (*n* = 200) of isolates subjected to TS and having a variety of Shiga toxin profiles were also sub-typed by PFGE using *Xba*I restriction according to the standard 1-d protocol [23]. Isolates were selected from serogroups O26 (*n* = 71), O103 (*n* = 62), O111 (*n* = 17) and O157 (*n* = 50) and were typed by PFGE using a CHEF DR II electrophoresis unit (Bio-Rad Laboratories, Mississauga, ON, Canada). Banding patterns were viewed with UV illumination and photographed using the Speedlight Platinum Gel Documentation System (Bio-Rad). Pulsed-field gel electrophoresis patterns were classified as unique or grouped in clusters with 90% or greater similarity as determined using Dice similarity coefficients, unweighted pair group methods arithmetic average algorithm, 1% position tolerance and 0.5% optimization (BioNumerics 7.6, Applied Maths NV, Sint-Martens-Latem, East Flanders, Belgium).

### 2.5. Comparing Consensus Sequences of PCR Primers and stx

Consensus between primers used (LAB A and LAB C) and *stx2* were analyzed by comparing primer sequences to *stx* gene sequences deposited within the NCBI database. For this, PCR primers from LAB A [10] and one set of primers used by LAB C [7] were evaluated using R10 Geneious software (Biomatters Inc., Newark, NJ, USA). Genetic information for individual reference subtypes (*stx2a-f*) were selected based on data by Scheutz et al. [24]. Using the Basic Local Alignment Search Tool (BLASTn), reference strains were compared to the NCBI database. All sequences with a similarity of >98% and >1200 nucleotides in size including A and B toxin gene subunits were aligned to reference strains with the alignment tool within the Geneious program at cost matrix (65% similarity). From the NCBI nucleotide collection a total of 357, 30, 357, 356, 30, 15 and 14 data sets were selected for the 2a-2g subtypes, respectively. The *stx2* primer sequences were tested and mapped versus aligned sequences.

### 2.6. Statistical Analyses

Correspondence between serogroup as determined by PCR and TS was compared using generalized linear mixed models (Proc Glimmix, SAS 9.3, SAS Institute Inc., Cary, NC, USA) with a binomial distribution and with O-group as a fixed effect. Model adjusted means (back-transformed to original scale) and 95% confidence intervals were reported, with *p* values < 0.05 deemed significant. To reduce discordance due to mixed cultures, frequency of agreement in *stx1* and *stx2* detection by serogroup was compared only for isolates where serogroup determined by PCR agreed with that determined by TS. In those analyses, O group and laboratory were fixed effects and a binomial distribution was used comparing Shiga toxin gene detection (always vs. intermittently detected). In instances where TS O-typing showed consistent (3 or more occasions) mismatching with PCR-based O-typing, the serogroup PCR primers were compared against TS-identified serogroup genes available in NCBI, using Geneious R10.

## 3. Results

### 3.1. O Group as Determined by PCR and TS

A total of 689 isolates were subjected to TS (Table 1). O group determined by PCR and TS showed less agreement (*p* < 0.05) for O26 and O45 as compared to other serogroups. Diversity within PCR-determined O26 and O45 was pronounced, with TS classifying each of these into more than 20 other O groups which were typed into more than 15 H groups. In contrast, 95% of O111 isolates were O111 by both PCR and TS, with the remainder not classifiable to an O group by TS (O?). Although the majority of O group mismatches between PCR and TS resulted in serotypes with only one or two isolates, 11.2% of PCR-determined O45 isolates were attributed to serotype O110:H31, while 2.8% of O157 isolates were attributed to O71:H32. However, analyses comparing sequences of *wzx* and *wzy* genes for O110 and O71 within the NCBI database to Conrad et al. [10]

PCR primers used found < 50% sequence identity, although neither O110 nor O71 are well-characterized within this database and non-specific binding could have occurred outside of the *wzx* or *wzy* genes.

### 3.2. Serotypes of Isolates Where PCR O Group Agreed with TS

For isolates where O group determined by PCR and TS agreed (*n* = 525), numbers of serotypes varied widely by serogroup (Table 2). Only a few serotypes were identified for O111 (*n* = 2), O145 (*n* = 3) or O157 (*n* = 4). In contrast, numerous serotypes were present for serogroups O103 (*n* = 15) and O45 (*n* = 14), with O26 (*n* = 6) and O121 (*n* = 9) being intermediate in this regard. Most prevalent serotypes per serogroup were: O26: non-motile (NM); O45:H4; O103:H2, O103:H21, O103:NM; O111:H8, O111:NM; O121:H7, O121:H19, O121:H46; O145:NM and O157:H7. A total of 45 isolates could not be O-typed by TS, the majority of which (*n* = 40) had an unidentifiable O group (O?), with the remaining untypeable isolates (*n* = 5) classed as O rough (OR).

### 3.3. Comparison of PFGE and TS for O26, O103, O111 and O157

A subset of isolates (*n* = 200) were subjected to both PFGE and TS and included 37 isolates where O group as determined by PCR and TS did not match and 12 isolates that were not typeable by TS (Table 3). Clustering of isolates which exhibited a minimum of 90% similarity based on PFGE analyses varied by serogroup, ranging from 12 clusters for O26 and O103 to 4 for O111. For isolates where serogroups determined by PCR and TS were in agreement (*n* = 163), those of O26 did not exhibit a high degree of similarity using PFGE, as 40.4% were considered unique. Although 37.9% of O103 were also classified as unique isolates by PFGE, 4 of the O103 serotypes evaluated consisted of only single isolates while isolates of the more common serotypes O103:H8 and O103:H2 were generally part of serotype-specific clusters. The majority of O157:NM and O157:H7 isolates were also part of serotype-specific clusters. Isolates of O157:H12 and O157:H29 were classified by PFGE as unique, although only two isolates of each of these serotypes were evaluated. Clustering of O111 showed an almost perfect correspondence with serotype, including O111:NM where 5/6 were present in clusters exclusive to O111:NM. In contrast, <50% of NM isolates for O26 and O103 were present in serotype-specific clusters.

### 3.4. Inconsistent Detection of stx1 and stx2

Comparing only isolates where O group agreed by PCR and TS, differences were also noted in the consistency whereby *stx1* and *stx2* were detected by the three laboratories within specific serogoups (Table 4). Overall, detection of *stx* varied most between LAB A and LAB C, with results for LAB B intermediate between the two extremes. Across labs, *stx1* was most consistently identified (*p* < 0.01) in O111 and O157:H7/NM isolates. In contrast, 80.6% of O45 isolates were positive for *stx1* at initial isolation in LAB A, while no *stx1* was detected in O45 by LAB C. *Stx2* was detected at LAB C only in isolates of O145, O121 and O157:H7/NM, while at LAB B *stx2* was also detected in isolates of O26 and O157:H12/H29. In contrast to other laboratories, *stx2* was detected by LAB A in isolates from all serogroups. Comparing labs, classification of O103 isolates as STEC varied by more than 25 fold from LAB A (69.8%) to LAB C (2.8%), with LAB B being intermediate (41.2%). Serotype of O157 profoundly affected classification as STEC, with 93.2% of O157:H12/H29 STEC at initial isolation by LAB A compared to 0% at LAB C. In contrast, proportion of O157:H7/NM isolates which were STEC varied by <10% across laboratories, ranging from 98.8 (LAB B) to 91.4% (LAB C).

Only a few specific serotypes were classified as STEC by LAB A and LAB C (Table 4). In contrast to other serogroups with two or fewer STEC serotypes detected at these laboratories, O121 had four different serotypes that were consistently STEC, although two were uncommon and represented by only one or two isolates. Similarly, serotypes O103:H11 and O103:H25, while STEC at LAB A and LAB C were represented by three or less isolates. Serotypes with at least eight isolates which were STEC at LAB A and LAB C included O26:H11, O111:H8, O111:NM, O121:H7, O121:H19, O145:NM, O157:H7 and O157:NM. Additional serotypes were STEC at both LAB A and LAB B included O26:H9, O26:H18, O26:H32, O26:H46, O26:NM, O103:H2, O103:H8, O103:H19, O103:H21, O103:H38, O103:NM, O157:H12, and O157:H29, although some of these classifications were based on less than three isolates.

**Figure 1:**
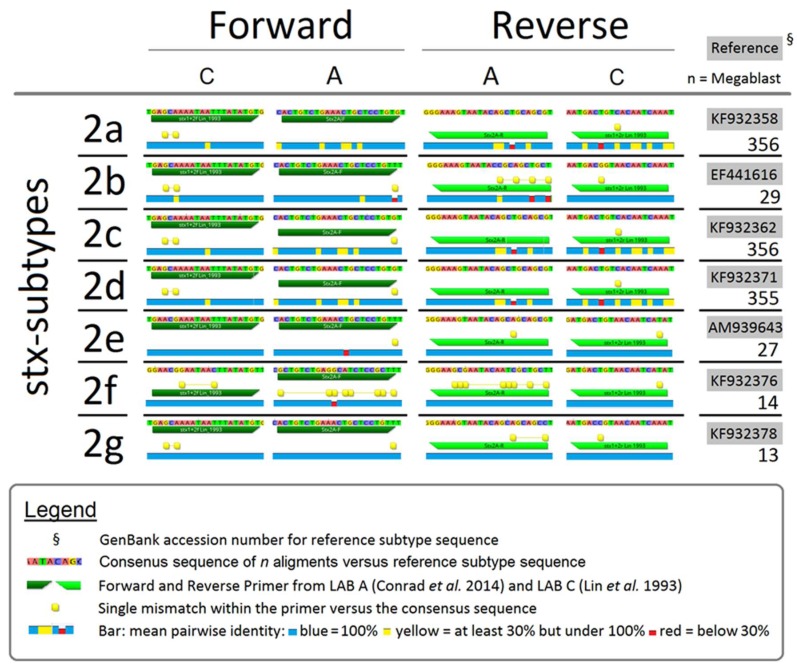
Comparison of PCR primers used by LAB A, Conrad et al. [10] and LAB C, Lin et al. [7] for consensus with sequences of *stx2* subtypes.

Comparison of Conrad et al. [10] (LAB A) and Lin et al. [7] (LAB C) primers versus the consensus sequences of multiple *stx2* reference subtypes (Figure 1), revealed mismatches between primers and consensus sequences. Therefore, LAB A was unlikely to detect *stx2b* or *stx2f*, which was confirmed by Conrad et al. [10] primers failing to amplify PCR amplicons from DNA of reference strains for these *stx2* subtypes. Even with one or two mismatches in forward and/or reverse primers, Conrad et al. [10] primers yielded positive amplification of *stx2* subtypes 2a, 2c, 2d, 2e, and 2g. However, even if primers matched a number of subtype sequences, hyper-mutable regions with <30% sequence identity were located within targets for the majority of *stx2* subtypes in both of the Lin et al. [7] and Conrad et al. [10] primers.

Impact of serotype on detection of *stx* was further assessed by repeating PCR analyses for 2 key STEC serotypes at LAB A using the same PCR methodology as in the initial isolation, but after an interval of up to four-year storage of isolates in glycerol at −80 °C. Isolates of O121:H19 (*n* = 12) remained stable for *stx2* between the initial isolation, LAB C analyses and for the repeat PCR analysis by LAB A (Table 5). However, multiple permutations were noted for *stx1* detection, including changes from positive to negative and vice versa even if only analyses by LAB A were considered. In contrast to O121:H19 that showed stability of *stx2*, isolates of O111:H8/NM (*n* = 6) showed stability of *stx1* over time and among all three laboratories, while *stx2*, if present, was only detected in the initial isolation (Table 6).

## 4. Discussion

### 4.1. Determination of O Group by PCR and TS

As isolates were picked directly from a plate after IMS without further purification, the presence of mixed cultures most likely contributed to different O-typing by PCR and TS. Cross-reactivity of serogroup-specific IMS kits for non-target serogroups has also been identified as a factor that can lead to variability in serogroup identification [4,5]. Another potential explanation would be cross-reactivity of PCR primers although the instances in the present study where the same mismatch occurred three or more times (O110:H31 for O45 and O71:H32 for O157) were not due to sequence identity between primers and *wzx* or *wzy* genes for these serogroups. Of 184 known *E. coli* O serogroups, Iguchi et al. [25] found that 145 had unique O-antigen gene clusters, 37 shared identical or very similar O antigen genes, while serogroups O14 and O57 did not contain O antigen genes at typical loci. More importantly, *wzx* and *wzy* genes for Top 7 are thought to have unique sequences [16,25], reducing the likelihood of cross-reactivity among PCR primers used in the present study.

Discrepancies between molecular methods and TS have been previously reported [26] as has cross-reactivity in antibodies used in TS [14,27,28]. However, heightened discrepancies between TS and PCR for O26 and O45 are puzzling when compared to other serogroups. A lack of selectivity by antibodies used in IMS is more prevalent for O111 compared to other Top 7 [5,9], but in the present study, O groups determined by PCR and TS were in agreement 95% of the time for O111. Strains of O26:H11 have been shown to undergo frequent rearrangements in their chromosome, virulence plasmids and pathogenicity islands [29]. Perhaps these traits are serogroup-wide and if so, could potentially have contributed to the inconsistencies in identification in the present study. Compared to O26, O45 has been linked to fewer cases of human disease [1,29], and the extent of plasticity of the O45 genome has been less studied.

### 4.2. Serotypes of Top 7 Isolated from Cattle Feces

Although the population of *E. coli* in the gastrointestinal tract of cattle is known to be diverse [22,30], relatively few STEC serotypes have been diagnosed as responsible for the majority of human disease, including O26:H11, O45:H2 O103:H2, O111:H8, O121:H19, O145:H26, O157:H7 and their non-motile forms [29]. Bosilevac and Koohmaraie [1] suggested that O103:H11, O103:H25 and O121:H7 should also be included in this list. Of this larger group, the only serotypes not detected in western Canadian cattle feces in the present study were O45:H2 and O145:H26, possibly due to geographical origin of isolates impacting local genetic variation [31]. O103 is among the most common serogroups isolated from cattle feces in North America [22,32] and along with O45 showed the highest diversity in serotypes. Diversity in O103 H antigens in isolates from livestock and human infections has also been documented [33,34].

Some studies of serotype diversity have not included O45 [35], likely due to an overall lower incidence of human disease associated with this serogroup compared to other members of the Top 7. Less study of O45 likely explains why one of the previously unreported *E. coli* serotypes identified in our study was from this serogroup (O45:H34). Other previously unreported serotypes included O103:H52 and O121:H32. Detection of O121:H32 would only be possible by TS due to the cross-reactivity in PCR analyses between O121 *fliC*_H19_ and *E. coli fliC*_H32_ isolates noted by Beutin et al. [36] which may lead to the misidentification of O121:H32 as O121:H19.

Only two serotypes of O111 were identified: O111:H8 and O111:NM. Molecular serotyping or whole genome sequencing would be necessary to determine if O111:NM strains were genetically O111:H8 that had one or more non-functional genes associated with motility, although other serotypes such as O111:H2 and O111:H10 have been associated with cattle and humans [35]. Compared to other serogroups, a lower number of O111 isolates were collected and the lack of diversity may be due to low numbers of isolates obtained. This may reflect difficulties in isolation of more diverse serotypes of O111, although challenges in isolation of Top 7 as yet have only been described based on serogroup [4,22]. Two serogroups showing little diversity in serotype (O145 and O157) are thought to have shared a common enteropathogenic *E. coli* ancestor before evolving further [37], although Eichhorn et al. [35] proposed that typing H antigens more accurately describes the phylogeny of STEC than does O group characterization. Accordingly, Iguchi et al. [25] suggested that STEC strains with the same H antigen such as O26:H11 and O111:H11 may be more closely related than strains with a common O-antigen. In the present study, the most prevalent H antigens found were H7 (O103, O121, O157); H8 (O45, O103, O111); H11 (O26, O45, O103); H14 (O45, O103, O121) and H19 (O45, O103 and O121).

### 4.3. Impacts of Serogroup and Serotype on PFGE Analyses

From PFGE analyses, serotypes associated with severe human disease [O103:H2, O111:NM, O111:H8, O157:NM and O157:H7,29] showed heightened genetic similarity and were more likely to be grouped within a serotype-specific cluster than were serotypes less-associated with human disease. The exception was O26:H11, which showed little genetic homogeneity, possibly due to the frequent genetic rearrangements that have been noted to occur in isolates of this serotype [38]. Increased genetic diversity among NM isolates would be expected as the majority of these strains are PCR-positive for flagellum genes but cannot be H-typed using motility test media [39]. Accordingly, most NM strains of O26 and O103 did not cluster with other NM isolates of their serogroup. In contrast to O26 and O103, NM strains of O111 and O157 were generally in serotype-specific clusters. Possibly, O157:NM or O111:NM strains could share a common mutation affecting flagellum assembly or motility, or carry a genetically-similar cryptic flagellum gene [40], which would result in O157:NM and O111:NM-specific clusters.

The most striking differences among serogroups revealed by PFGE analyses were the distinct characteristics of O26 as compared to O103, O111 and O157. In the latter three serogroups, numbers of clusters increased in proportion with the numbers of isolates evaluated and serotype-specific clusters were present. In contrast, serotypes of O26 isolates showed little relation to clusters and numbers of clusters and unique isolates were equal to that of O103, even though 40% fewer O26 isolates were included in these analyses. Accordingly, additional study is clearly warranted of genetic rearrangements of serotypes of O26 other than O26:H11.

### 4.4. Inconsistent Detection of stx1 and stx2

Although detection of Shiga toxins was evaluated only for isolates where O group by PCR matched that determined by TS, mixed cultures would have been responsible for some of the inconsistent detection of Shiga toxin genes by PCR noted in the present study. If cultures were mixed, proportions of different serogroups of *E. coli* have been shown to change markedly during incubation [41], possibly affecting Shiga toxin detection when a single colony is later picked for evaluation. However, factors in addition to mixed cultures would have likely contributed to inconsistent detection of *stx* in the present study.

A possible reason for differences in *stx* detection rates between laboratories would be the primers used for PCR analysis. Comparing primer targets used by LAB A and LAB C against *stx2* subtype sequences within the NCBI database showed that mismatches existed for both primer sets. It was previously shown that the primers used by LAB A [10] were unable to detect *stx2b* or *stx2f* due to multiple mismatches between the primers and the *stx2* consensus sequences (Figure 1). Primer sets used by LAB A and LAB C both showed strengths and weaknesses, with mismatches also varying according to hyper-mutable regions within *stx2* subtypes. In contrast, a database analysis revealed that *stx1* coding sequences are highly conserved and as a result, the different *stx1* primer systems used by LAB A and LAB C consistently detected target genes.

Differences in detection of *stx* based on PCR primers were comprehensively evaluated by Ziebell et al. [12] who concluded that nucleotide sequence variations in primer binding sites were frequently responsible for failure to detect *stx* by the protocols evaluated. However, even if primer sequences perfectly match targets, PCR amplifications are influenced by varying efficiencies of the different stages (denaturing, annealing and elongation) of every cycle [42], which were not compared in the present study. Due to rapid evolution of STEC [43], additional studies are required to re-evaluate PCR primer binding sites to ensure continued effective surveillance of foodborne pathogens. Even when the same PCR primers and DNA template sources are employed, variation in *stx1* and *stx2* detection has occurred across laboratories with this variation attributed to differences in reagents and thermocyclers [24], highlighting the potential difficulty in comparing results for STEC detection across laboratories.

Even with the possibility of mixed cultures and effects of PCR methodology, other factors are necessary to possibly explain differences in identification of serogroup O45 isolates, where 80.6% of were identified as STEC upon initial isolation, whereas none were identified as STEC when serotyped. Shiga toxin genes are located in bacteriophages (phages) integrated into the host genome or present as free phages in the environment [44,45,46]. For initial detection of STEC by molecular methods, Quiros et al. [21] recommended a filtration step which eliminated more than 99% of free phage particles and increased the efficiency and reliability of PCR for STEC detection. As a filtration step was not performed in the present study, the presence of free *stx* phage [47] may have resulted in overestimation of STEC at the initial isolation. However, even when *stx*-phages are integrated into the bacterial genome, they are considered mobile genetic elements [44] and the possibility of treating an established STEC infection by inducing virulence gene deletion in the colonizing bacteria has been suggested [43].

A total of 7% of O45 isolates at LAB C were positive for *eae* and *hylA* and were considered atypical enteropathogenic *E. coli*. Enteropathogenic strains are also a public health concern [48] but would be overlooked by protocols which rely on detection of *stx* as the initial screening step. Due to potential for integration and loss of *stx* phages as well as genetic and phenotypic similarities among STEC O157:NM and Shiga-toxin negative enteropathogenic strains, Ferdous et al. [49] questioned the reliability of classifying *E. coli* based on detection of *stx* and *eae*. Genetic changes occur rapidly in *E. coli* after initial environmental isolation during multiple rounds of cultivation in nutrient-rich media, a process termed domestication [50]. Accordingly, Joris et al. [19] documented the loss of *stx1* and *stx2* as determined by PCR in isolates of O26, O103 and O157 after the first sub-culture and found that loss of these toxin genes was rare in O157 as compared to non-O157 strains. In the present study, we found minimal changes in detection of *stx* in isolates of O157:H7/NM. In contrast, 93.2% of O157:H12/H29 were STEC at initial isolation, 42.9% were STEC at LAB B and 0% STEC at LAB C, although it is important to note that Shiga toxins have not previously been reported in O157:H12 or O157:H29 [51,52]. As these latter two serotypes are less common than H7/NM, it is possible that the Joris et al. [19] study group of 20 isolates consisted of only H7/NM. A greater stability of *stx2* in isolates of O157:H7 as compared to O157:NM has been previously documented [53], an outcome that might be due to differences in the integration sites for *stx2* phages [45].

Negative changes in *stx* detection noted in the present study were considerably higher than the maximum 20% loss reported by Joris et al. [19] likely a reflection of differing PCR methodologies used by the three laboratories in the current study. Joris et al. [19] used the method of Possé et al. [54] for initial bacterial isolation which included novobiocin, vancomycin, rifampicin, bile salts and potassium tellurite in the media. Sub-therapeutic doses of a variety of antibiotics have been shown to induce a lytic cycle in *Stx*-phages [44,55] and care was taken by all laboratories in the present study to avoid use of antibiotics or substances like potassium tellurite in media, a practice that can bias isolation of non-O157 *E. coli* [56]. As antibiotic use in media did not influence *stx* detection by the three laboratories, the use of the same DNA template for approximately 50% of the isolates evaluated by LAB B likely increased the similarity of PCR results between LAB B and LAB A.

For isolates of O111, detection of *stx1* was stable across laboratories and not influenced by time. The *stx1* phage of O111 is thought to be cryptic due to a lack of integrase and exicisionase genes [57], although Asadulghani et al. [58] determined that prophages with multiple genetic defects could excise themselves, replicate and be released from strains of O157. The stability of *stx1* in O111 isolates was in contrast to *stx2* which was only detected at initial isolation and was not detected in repeat PCR analysis using sub-cultured isolates by LAB A. Deletion of *stx2* phage from strains of O111:H8 has been observed in patients during an outbreak in Japan and during in vitro incubations of these isolates [59]. These authors speculated on a highly dynamic system that can result in both the acquisition and loss of *stx2* phages. While results of the present study did not demonstrate acquisition of *stx2* in O111, it did occur with *stx1* in O121:H19 (Table 5).

The stability of *stx2* in isolates of O121:H19 in the present study as compared to the loss or acquisition of *stx1* on repeat PCR analyses is interesting. Changing *stx1* detection may point to differences in insertion sites for the *stx1* and *stx2* phages of O121:H19 or may be related to an uncharacterized insertion sequence element in *stx1* as previously documented [20] or proposed for *stx2* [24]. In Canada, O121:H19 has been recently implicated in human disease outbreaks due to contaminated flour [60] and it is also likely not coincidental that serotypes of *E. coli* frequently implicated in human disease are those that show stability in *stx1* and/or *stx2* across laboratories and over time. O121 also showed the most diversity in STEC serotypes (*n* = 4) detected upon initial isolation at LAB A and at LAB C. To our knowledge, one of these serotypes (O121:H32), has not been previously reported, and may be an emerging pathogen as the majority of serotypes in the present study with stable *stx* have been linked to human disease.

## 5. Conclusions

**Figure 2:**
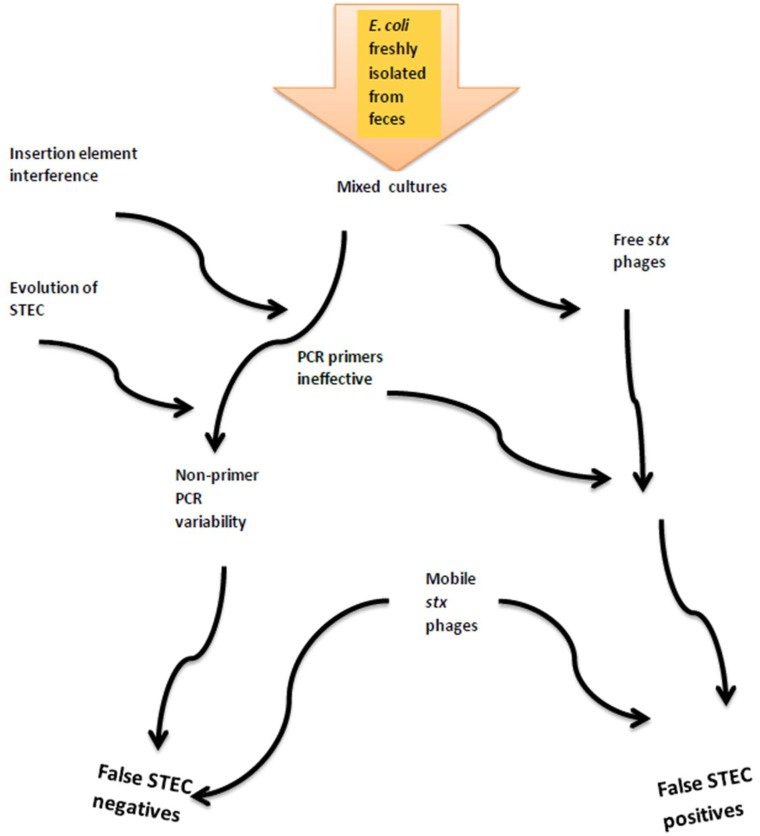
Factors contributing to cumulative errors in detection of Shiga toxin genes and classification of isolates as STEC.

The detection of STEC and pathogenic microorganisms in general represents an ongoing global challenge to ensure food safety along the farm-to-fork continuum as well as in human medicine. Pathogen surveillance is based on molecular and microbiological methodologies, but as STEC genomes continue to evolve, surveillance methods require adaptation and refinement. In addition, some microbial adaptations may coincide with weaknesses in existing diagnostic tools and prevent the identification of pathogens and/or virulence factors. Using detection of *stx* in cultures or isolates as the initial screening would ignore atypical enteropathogenic *E. coli* and also due to loss or gain of *stx* phages may underestimate risks to human health. In the absence of uniform assays, monitoring and characterizing STEC can vary substantially across laboratories as demonstrated in the present study. Combined, we showed that multiple factors relating to lab diagnostics and bacterial evolution could possibly affect characterization of STEC (Figure 2). New or improved methods that can be routinely and rapidly applied across multiple laboratories are required for conclusive and consistent identification of pathogenic *E. coli.* With continually reducing costs and increasing speed, next-generation sequencing may at some time in the future meet at least some of these needs.

## Figures and Tables

**Table 1:** Differences between PCR and traditional serotyping (TS) for O group determination among serogroups.

PCR-O Group	#Isolates Serotyped	Isolates PCR = TS O Group (%)	SEM ^1^	Predominant Mismatches (#of Isolates)	#Mismatched O Groups by Serotyping	#H Groups by TS of Mismatched O Groups
O26	115	62.6 ^a^	4.5	NA ^2^	34	22
O45	116	69.8 ^a^	4.3	O110:H31 (13)	21	16
O103	116	89.0 ^b^	2.9	NA	8	6
O111	39	95.0 ^b^	3.5	NA	1	1
O121	115	91.4 ^b^	8.6	NA	9	9
O145	79	92.4 ^b^	7.6	NA	6	5
O157	109	83.9 ^b^	3.5	O71: H32 (3)	13	9
Total	689	83.0	−	NA	92	68

^a,b^ Means in a column with different superscripts differ (*p* < 0.05). ^1^ SEM, standard error of the mean. ^2^ NA, not applicable as mismatches were not repeated (*n* < 3 per mismatch). ^a,b^ Means in a column with different superscripts differ (*p* < 0.05).

**Table 2:** Numbers of Top 7 isolates of each serotype including only those where PCR and traditional serotyping determined the same O group (*n* = 525).

		O Group							
		**O26**	**O45**	**O103**	**O111**	**O121**	**O145**	**O157**	TOTAL
H group by traditional serotyping	NM ^1^	42	3	23	21	6	64	11	**170**
	7			4		54		55	**126**
	4		34						**34**
	19		6	2		20			**28**
	2			26					**26**
	21			18		1			**19**
	46	1				16			**17**
	9	5	8						**13**
	11	9	1	2					**12**
	12							11	**11**
	10		7			3			**10**
	38		1	9					**10**
	29							8	**8**
	14		2	4		1			**7**
	unknown		2	2		2	1		**7**
	8		1	5	13				**6**
	32	3				1			**4**
	16		1	2					**3**
	25			1			2		**3**
	6			2					**2**
	18	2							**2**
	30		2						**2**
	34		2						**2**
	43			1					**1**
	45		1						**1**
	52			1					**1**
	TOTAL	**62**	**71**	**102**	**34**	**104**	**67**	**85**	**525**

^1^ NM, not motile. Yellow highlighted cells show most common serotypes per O group. Green highlights identify the most common H antigens across O groups. Blue highlighted cells shows serotypes previously unreported in cattle feces.

**Table 3:** Correspondence between traditional serotyping (TS) and pulsed field gel electrophoresis of isolates of O26 (*n* = 71), O103 (*n* = 62), O111 (*n* = 17), and O157 (*n* = 50) initially serogrouped by PCR.

Serogroup	#Clusters ^1^	#(%) NT ^2^ Isolates	#(%) PCR Mismatch TS Serogroup	#PCR and TS Matching Isolates	Isolates Where PCR and Traditional Serotyping Matched Serogroup #Isolates Within Serogroup-Specific Clusters #Unique Isolates							
					O26:NM	O26:H9	O26:H11	O26:H32				
O26	12	5(7.0)	29 (40.8)	42	11/23	1/3	2/6	1/3				17 (40.4)
					O103:NM	O103:H2	O103:H6 ^3^	O103:H7	O103:H8	O103:H14	O103:H19	
O103	12	2(3.2)	2 (3.2)	58	4/14	8/11	1/1	0/3	4/4	2/4	0/1	22 (37.9)
					O103:H21	O103:H25	O103:H38	O103:H43				
					6/10	0/1	2/6	0/1				
					O111:NM	O111:H8						
O111	4	3(17.6)	0 (0)	14	5/6	8/8						0 (0)
					O157:NM	O157:H7	O157:H12	O157:H29				
O157	7	2(4.0)	6(12.0)	42	3/5	24/29	0/2	0/2				9 (21.4)

^1^ Clusters where isolates show a minimum of 90% similarity. ^2^ NT, not typeable by traditional serotyping. ^3^ Isolate clustered with non-motile (NM) isolate.

**Table 4:** Detection of virulence genes at initial isolation (LAB A), LAB B and LAB C.

O Group	#Isolates Labs A and C (LAB B)	% STEC ^1^ LAB A	% STEC LAB B	% STEC LAB C	%*stx1* + Labs A&B	% *stx1* + Labs A&C	%*stx2* + Labs A&B	% *stx2 +* Labs A&C	H Types STEC in Labs A& C (n)
O26	62 (52)	71.5 ^a^ ± 3.2	30.0 ^a^ ± 3.2	8.3 ^a^ ± 3.0	25.5 ^a^ ± 2.9	8.3 ^b^ ± 3.3	3.6 ^a^ ± 1.3	0.0 ^a^ ± 0.0	H11 (7/9)
O45	71 (0)	80.6 ^ab^ ± 2.8	NE ^1^	0.0 ^a^ ± 0.0	NE	0.0 ^a^ ± 0.0	NE	0.0 ^a^ ± 0.0	None
O103	102 (31)	69.8 ^a^ ± 3.0	41.9 ^a^ + 7.5	2.8 ^a^ ± 1.5	39.5 ^b^ ± 2.3	2.8 ^a^ ± 1.6	0.0 ^a^ ± 0.0	0.0 ^a^ ± 0.0	H11 (2/2), H25 (1/1)
O111	34 (15)	100.0 ^c^ ± 0.0	100.0 ^b^ + 0.0	89.5 ^c^ ± 4.5	100.0 ^c^ ± 0.0	89.5 ^d^ ± 4.5	0.0 ^a^ ± 0.0	0.0 ^a^ ± 0.0	H8 (21/21), NM (9/13)
O121	104 (0)	85.2 ^b^ ± 2.7	NE	33.0 ^b^ ± 4.2	NE	15.1 ^b^ ± 3.5	NE	16.0 ^b^ ± 3.6	H7 (16/54), H10 (2/3) H19 (15/20), H32 (1/1)
O145	67 (0)	80.8 ^ab^ ± 4.5	NE	49.3 ^b^ + 5.3	NE	49.3 ^c^ ± 5.9	NE	2.7 ^a^ ± 1.9	NM (32/64)
O157:H12/H29	19 (19)	93.2 ^c^ ± 2.2	44.2 ^a^ ± 7.6	0.0 ^a^ ± 0.0	42.9 ^b^ ± 7.7	0.0 ^a^ ± 0.0	21.4 ^b^ ± 5.9	0.0 ^a^ ± 0.0	None
O157:H7/NM	66 (54)	98.7 ^c^ ± 1.4	98.8 ^b^ ± 1.2	91.4 ^c^ + 3.0	81.6 ^c^ ± 3.0	80.0 ^d^ ± 4.8	84.3 ^c^ ± 2.7	90.7 ^c^ ± 4.2	H7 (54/55), NM (7/11)

^a,b,c,d^ Means in a column with different superscripts, differ (*p* < 0.01). ^1^ NE, not evaluated. ^1^ STEC, positive for *stx1* and or *stx2*. Includes only isolates (*n* = 525) where O group was the same by PCR and TS, with a subset of these (*n* = 171) evaluated by LAB B. All LAB A isolates were sub-cultured and transported on slants to LAB C.

**Table 5:** Changes in carriage of *stx1* and consistent detection of *stx2* in twelve isolates of O121:H19 from PCR analysis by two labs.

#Isolates Sharing PCR Profile	PCR	Year of PCR	LAB	PCR *stx1*	Detection ^2^ *stx2*	*Comments*
6	First ^1^	2013	A	+	+	LAB A PCRs are the same over years, LAB C negative for *stx1*
	Second	2015	C	-	+	
	Third	2017	A	+	+	
2	First	2013	A	-	+	All three PCRs are the same never *stx1*
	Second	2015	C	-	+	
	Third	2017	A	-	+	
2	First	2013	A	-	-	LAB A and LAB C initially the same, *stx1* positive in second LAB A analysis
	Second	2015	C	-	-	
	Third	2017	A	+	-	
2	First	2013	A	+	+	Second LAB A analysis negative for *stx1* and now agrees with LAB C
	Second	2016	C	-	+	
	Third	2017	A	-	+	

^1^ PCR first = initial isolation. ^2^ PCR detection + = positive, - = negative.

**Table 6:** Changes in carriage of *stx2* and consistent detection of *stx1* in six O111:H8/NM isolates from PCR analyses by three laboratories.

#Isolates Sharing PCR Profile	PCR	Year of PCR	LAB	*PCR* *stx1*	Detection ^2^ *stx2*	Comments
4	First ^1^	2013	A	+	−	*stx2* never detected by any laboratory
	SecondThird	2014 2014	B C	+ +	− −	
	Fourth	2017	A	+	−	
2	First Second	2013 2014	A B	+ +	+ −	*stx2* present in initial isolation PCR only
	Third	2014	C	+	−	
	Fourth	2017	A	+	−	

^1^ PCR first = initial isolation. ^2^ PCR detection + = positive, - = negative.
